# Serum Vitamin B12 and Homocysteine Levels: Correlation with MRI Findings and Cognitive Performance in a Rural South Indian Cohort

**DOI:** 10.1002/alz70862_110226

**Published:** 2025-12-23

**Authors:** Sulakshna Aggarwal, Priya Chatterjee, Hitesh Pradhan, Jonas S Sundarakumar

**Affiliations:** ^1^ Centre for Brain Research, Bangalore, Karnataka India; ^2^ Centre for Brain Research, Indian Institute of Science, Bangalore, Karnataka India

## Abstract

**Background:**

Vitamin B12 is known to influence cognitive functioning potentially through the influence on the serum levels of homocysteine. There is a dearth of data assessing the association of Vitamin B12 and Homocysteine with MRI brain volumes and cognitive performance in the rural Indian population.

**Method:**

We used baseline cross‐sectional of the non‐demented (Clinical Dementia Rating, CDR score<1) participants aged 45+ years (*n* = 2784) from the CBR‐SANSCOG (Centre for Brain Research‐Srinivaspura Aging NeuroSenescence and COGnition) study cohort. Cognitive performance was assessed using Hindi Mental State Examination (HMSE) test and the culturally adapted computerized COGNITO (Computerized assessment of adult Information processing) battery. The domain‐specific (attention, memory, language, visuospatial ability) cognitive scores were standardized and a composite score was calculated from the weighted averages of the individual test scores. General linear model was used to assess the association between serum vitamin B12, homocysteine levels and the cognitive performance adjusting for age, sex, education, tobacco, alcohol use, hypertension, diabetes, and depression. Using 3T MRI, Region of Interest(ROI) based‐analysis was done to study the association between vitamin B12, homocysteine, and MRI brain volumes normalized for total intracranial volume (*n* = 1268), adjusting for age, sex, and education.

**Result:**

A total of 36.5%, 34.3%, and 29.3% of the participants had deficient (<200pg/ml), insufficient (200‐300pg/ml), and sufficient vitamin B12 (>300pg/ml) levels, respectively, whereas 56% and 44% had normal (5‐15µmol/l) and high (>15µmol/l) levels of homocysteine, respectively. No association was found between vitamin B12 and the cognitive performance tests or any brain volumes. Increasing homocysteine levels were associated with decreasing scores on HMSE [β=‐0.02,(‐0.03,‐0.01), *p* = 0.02], visual attention [β=‐0.01,(‐0.02,‐0.00), *p* = 0.02] and hippocampal volume [β=‐0.01,(‐0.01,‐0.00), *p* = 0.02]. Homocysteine did not have a mediating effect between vitaminB12 and cognitive performance or any of the brain volumes.

**Conclusion:**

Increasing serum homocysteine levels were negatively associated with cognitive performance irrespective of serum vitamin B12 levels in this rural aging Indian population. Therefore, early screening for serum homocysteine could potentially flag individuals at risk for cognitive impairment and thereby, contribute to developing a multimodal composite biomarker for early detection of cognitive impairment.

